# Astragalus Polysaccharide Nanoemulsion: A Promising Adjuvant for Foot-And-Mouth Disease Virus-Like Particle Vaccines

**DOI:** 10.1155/tbed/6693841

**Published:** 2025-09-25

**Authors:** Xiaoni Shi, Zhidong Teng, Kun Yang, Hetao Song, Yun Zhang, Shuzhen Tan, Hu Dong, Shiqi Sun, Yaozhong Ding, Huichen Guo

**Affiliations:** ^1^State Key Laboratory for Animal Disease Control and Prevention, College of Veterinary Medicine, Lanzhou University, Lanzhou Veterinary Research Institute, Chinese Academy of Agricultural Sciences, Lanzhou, China; ^2^School of Chemical Engineering, Lanzhou City University, Lanzhou 730070, China; ^3^College of Veterinary Medicine, Gansu Agricultural University, Lanzhou, China; ^4^Yunnan Tropical and Subtropical Animal Virus Diseases Laboratory, Yunnan Animal Science and Veterinary Institute, Kunming, Yunnan, China

**Keywords:** adjuvant, foot-and-mouth disease, nanoemulsion, virus-like particles

## Abstract

Vaccine immunization is the most cost-effective way for preventing infectious diseases, and the development of safe and effective adjuvants is crucial for ensuring vaccine efficacy. Due to the advantages of high safety profile, excellent stability, and significant immune-enhancing properties, nanoemulsions have become widely used adjuvants in animal vaccines. In this study, a novel astragalus polysaccharide nanoemulsion (APSN) was developed using pseudo-ternary phase diagram method combined with phase inversion technique. The resulting nanoemulsion exhibited a hydrated diameter of approximately 78.82 nm, with favorable stability and biocompatibility. A vaccine based on FMDV virus-like particles (VLPs) was formulated using APSN as an adjuvant and was used to immunize mice and pigs. Mouse immunization results demonstrated that APSN significantly enhanced the levels of specific antibodies, IgG1, IgG2a, IFN-γ, and IL-4 induced by FMDV VLPs. Comparing with ISA-206, immunization in pigs showed that APSN paired with FMDV VLPs induced higher levels of specific antibodies, neutralizing antibodies, IL-1β, IL-4, and IFN-γ. The above results indicate that APSN is a new type of nanoemulsion adjuvant with strong potential to enhance vaccine immunogenicity, contributing valuable insights to the development of nanoadjuvant-based vaccine formulations.

## 1. Introduction

Infectious diseases pose a significant threat to both human health and the livestock industry, causing substantial economic losses and impacting global food security. Traditional inactivated and attenuated vaccines have been instrumental in controlling numerous infectious diseases. However, these approaches require high-level biosafety facilities for production and carry the potential risk of incomplete inactivation or reversion to virulence [1, 2]. Consequently, there is a growing need for safer and more cost-effective vaccine platforms. Peptide vaccines and virus-like particles (VLPs) have emerged as promising alternatives, offering advantages in safety, production scalability, and cost-effectiveness [3]. VLPs mimic the structure of native viruses without containing infectious genetic material, eliciting robust immune responses while minimizing safety concerns. The successful commercialization of VLP-based vaccines against human papillomavirus, hepatitis B virus, and hepatitis E virus highlights their potential [4].

Foot-and-mouth disease (FMD) is a highly contagious viral disease affecting cloven-hoofed animals, causing significant economic losses due to trade restrictions, reduced productivity, and animal welfare concerns. Vaccination remains the cornerstone of FMD control, particularly in developing countries [5]. FMDV VLPs, composed of viral structural proteins, have shown promise as safe and effective vaccine candidates, potentially replacing traditional inactivated vaccines [6, 7]. However, like most subunit vaccines, FMDV VLPs often require adjuvants to enhance their immunogenicity and induce protective immune responses. Vaccines typically comprise two components: the antigen and the adjuvant. The adjuvant plays a critical role in enhancing antigen immunogenicity, stimulating immune responses, and prolonging antigen release. Aluminum salts, the first adjuvants discovered, have long been used to potentiate vaccine efficacy. Despite the development of numerous adjuvants since the 1920s, many have not been licensed due to safety and tolerability concerns [8].

Oil emulsions, composed of water, oil, surfactant, and cosurfactant, are widely studied due to their ease of preparation, low cost, biocompatibility, and ability to create an antigen depot [9, 10]. Four oil emulsion adjuvants—MF59 (seasonal trivalent influenza vaccine), AS01 (herpes zoster and malaria vaccines), AS03 (influenza vaccine), and AS04 (hepatitis B and HPV vaccines)—have been licensed for human use, following aluminum salts [8, 11], demonstrating the significant value of this adjuvant class. Beyond human vaccines, oil emulsions are also extensively used in veterinary vaccines, notably in FMD vaccines, in which ISA206 and ISA201, produced by Seppic (France), serve as prominent examples [12, 13]. Nanotechnology has further revolutionized vaccine adjuvant development. Nanoemulsions (less than 200 nm), offering enhanced kinetic stability, can encapsulate antigens, improving their dispersibility and permeability. Studies have demonstrated that the uptake of nanoparticles by antigen-presenting cell (APC) is size-dependent, with smaller particles being more readily internalized [14, 15]. Thus, nanoemulsions offer advantages in enhancing antigen bioavailability. For example, transdermal administration studies have shown that 80 nm nanoemulsions penetrate the epidermis more effectively than larger particles [16]. Despite this potential, the number of commercially available nanoemulsion-based pharmaceuticals remains limited.

Traditional Chinese medicine (TCM), with its long history of use in Asian countries, possesses valuable immunomodulatory properties [17]. For instance, quassin enhances nitric oxide production [18], *Ganoderma lucidum* polysaccharide activates dendritic cells [19], and ginsenosides boost CD4^+^ T cell activity [20]. Incorporating immunoenhancers into the aqueous phase of nanoemulsions can further augment their adjuvant properties. For example, the use of Ophiopogon D in a nanoemulsion formulation, significantly enhanced both cellular and humoral immune responses to an antigen [21]. Astragalus polysaccharide (APS), a TCM ingredient with known immune-enhancing effects, exerts regulatory effects on both central and peripheral immune organs, including bone marrow, thymus, lymph nodes, spleen, and mucosal tissues [22]. APS activates macrophages, dendritic cells, CD4^+^ T cells, CD8^+^ T cells, and cytotoxic T lymphocytes [23, 24]. Supplementing vaccines with APS has been shown to improve immune responses against avian infectious bronchitis virus [25], FMD virus [26], and SARS-CoV-2 [27], highlighting its potential as an effective immune booster.

Therefore, this study aimed to develop and evaluate a novel APS nanoemulsion (APSN) as an adjuvant for FMDV VLP vaccines. We synthesized APSN using pharmaceutical white oil as the oil phase and APS as the aqueous phase, and characterized its physicochemical properties. We then formulated an FMDV VLPs-based vaccines adjuvanted with APSN (VLPs–APSN) and evaluated its immunogenicity in mice, and pigs. Our results demonstrate the potential of APSN as a safe and potent nanoemulsion adjuvant capable of enhancing the immune efficacy of FMDV VLPs vaccines, contributing to the advancement of nanoadjuvant technology and FMD vaccine development.

## 2. Materials and Methods

### 2.1. Screening of Blank Nanoemulsion Formulations

Medical-grade white oil was used as the oil phase. Span 80 and Tween 80 were selected as surfactants, with a mass ratio of 2:1. 1,2-Propanediol, polyethylene glycol 400 (PEG-400), and anhydrous ethanol were evaluated as candidate cosurfactants. Nonionized water served as the aqueous phase. The optimal surfactant and cosurfactant mixture (Smix) ratio was determined using the pseudo-ternary phase diagram method. Briefly, Smix mixtures were prepared at different mass ratios (1:1, 2:1, and 3:2). Pseudo-ternary phase diagrams for each Smix combination were then constructed by titrating with water at room temperature to identify the nanoemulsion region. Origin 6.0 software (OriginLab, Northampton, MA, USA) was used to plot the phase diagrams, and the optimal Smix ratio was determined based on the size of the nanoemulsion region.

### 2.2. Preparation of APSN

Based on the optimized blank nanoemulsion formulation, the base formulation for preparing APSN was determined. The specific preparation method is as follows: First, a certain amount of APS was weighed and dissolved in deionized water to prepare aqueous solutions with concentrations of 1, 5, 10, 15, 20, and 25 mg/mL. These APS aqueous solutions constituted the aqueous phase. Subsequently, according to the blank nanoemulsion formulation, specific quantities of oil, surfactant, and cosurfactant were mixed thoroughly to obtain the oil phase. At room temperature, the aqueous phase was slowly added dropwise to the oil phase under continuous magnetic stirring. The appearance of the emulsions formed with different concentrations of APS solutions was visually observed. APS solution concentrations that resulted in the formation of a transparent and homogeneous nanoemulsion were considered candidate concentrations.

### 2.3. Characterization of the APSN

The morphology of APSN was examined by transmission electron microscopy (TEM). APSN was diluted 1:100 and applied dropwise to a 300-mesh copper grid. Images were acquired using a Hitachi HT7700 TEM (Tokyo, Japan). The hydrated particle size and zeta potential of APSN were determined by dynamic light scattering (DLS) using a Malvern Zetasizer Nano ZS90 (Worcestershire, UK). The particle size of FMDV VLPs was also measured.

### 2.4. Stability of the APSN

The stability of APSN was evaluated through centrifugation, repeated freeze–thaw experiments, and storage at room temperature. First, the prepared APSN was centrifuged at 10,000 and 12,000 rpm for 30 min, respectively, and photographs were taken to observe whether the APSN had stratified or become turbid after centrifugation. Then, the APSN was subjected to three freeze–thaw cycles at −30 and 25°C, with each temperature maintained for 48 h per cycle. Photographs were taken after each freeze–thaw cycle. The particle size of the nanoemulsion was measured by DLS after each freeze–thaw cycle. Finally, the APSN was stored at room temperature for 360 days, and photographs were taken to observe whether stratification or turbidity occurred before and after storage. Additionally, the hydrated particle sizes of APSN after 360 days of storage was measured using DLS and compared with the hydrated particles size of the initially prepared APSN.

### 2.5. Biocompatibility of APSN

All animal experiments were approved by the Animal Ethics and Experimental Committee of Lanzhou Veterinary Research Institute, Chinese Academy of Agricultural Sciences. Female BALB/c mice (6–8 weeks old, 16–20 g) were intramuscularly injected with 100 µg of APSN (twice the immunization dose) or 100 µg of PBS (control group). On day 28 postinjection, mice were euthanized, and the heart, liver, spleen, lung, and kidney were collected and weighed. Organ-to-body weight ratios were calculated as an indicator of potential toxicity. Tissues were fixed in 4% paraformaldehyde, embedded in paraffin, sectioned, and stained with hematoxylin and eosin (H&E). Histopathological changes were assessed using a digital trinocular microscope (BA400Digital; Motic, Carlsbad, CA, USA). Blood samples were collected from mice 7 days after immunization with PBS or APSN. Serum was separated, and biochemical parameters (aspartate aminotransferase, creatine kinase, creatinine, and andtotal bilirubin) were analyzed to assess the potential effects of APSN on liver and kidney function.

### 2.6. Preparation and Stability Assessment of FMDV–VLPs–APSN Vaccine

FMDV VLPs were produced in *E. coli* and purified as previously described [7]. The APSN was emulsified with FMDV VLPs vaccine at a mass ratio of 1:1. The emulsified vaccine was then centrifuged for 5 or 15 min at each speed of 6000 and 12,000 rpm, respectively, and photographs were taken to observe the appearance of the vaccine.

### 2.7. Immunization of BALB/c Mice

Twenty-four healthy, specific pathogen-free female BALB/c mice (6–8 weeks old) were randomly divided into four groups (*n* = 6): (1) PBS control, (2) FMDV VLPs (50 µg), (3) FMDV VLPs (50 µg) + ISA-206 (commercial adjuvant, 50 µg), and (4) FMDV VLPs (50 µg) + APSN (50 µg). Mice were immunized via intramuscular injection. Serum samples were collected on 14, 28, and 42 days after-immunization. Specific antibody titers were measured using a liquid-phase blocking ELISA kit (LVR Biotechnology Co., Lanzhou, China). IgG1 and IgG2a antibody levels were determined by indirect ELISA. The IFN-γ and IL-4 level were also measured using ELISA kits (R&D Systems, Minneapolis, MN, USA).

All mouse experiments were performed in accordance with the Guidelines for the Care and Use of Laboratory Animals of the Lanzhou Veterinary Research Institute, Chinese Academy of Agricultural Sciences, and were approved by the Institute's Animal Ethics Committee (Approval No: LVRIAEC-2022-023).

### 2.8. Immune Effect in Pigs

Fifteen 9-week-old finishing pigs, seronegative for FMDV antibodies, were obtained from a conventional farm and housed in three separate rooms with ad libitum access to food and water. Pigs were randomly assigned to three groups (*n* = 5): (1) PBS control, (2) FMDV VLPs (50 µg) + ISA-206 (50 µg), and (3) FMDV VLPs (50 µg) + APSN (50 µg). Pigs were immunized intramuscularly. Blood samples were collected on 0, 7, 14, 28, 35, 42, 49, and 56 days after immunization. Specific antibody titers were measured using a liquid-phase blocking ELISA kit (Lanzhou Veterinary Research Biotechnology Co., China). Neutralizing antibody titers in pig serum were determined by the microneutralization test. The levels of IL-1β, IFN-γ, and IL-4 in the serum were measured using porcine ELISA kits (OmnimAbs, Alhambra, CA, USA) according to the manufacturer's instructions.

### 2.9. Statistical Analysis

All data were analyzed using SPSS 22.0 software (IBM Corp., Armonk, NY, USA). Results are presented as mean ± standard deviation. Differences between two groups were assessed using the independent samples *t*-test. Differences among multiple groups were analyzed using one-way analysis of variance (ANOVA), followed by either the least significant difference (LSD) test or Dunnett's T3 test, depending on the homogeneity of variances. Differences were considered statistically significant at *⁣*^*∗*^*p* < 0.05.

## 3. Results

### 3.1. Synthesis and Characterization of APSN

**Figure 1:**
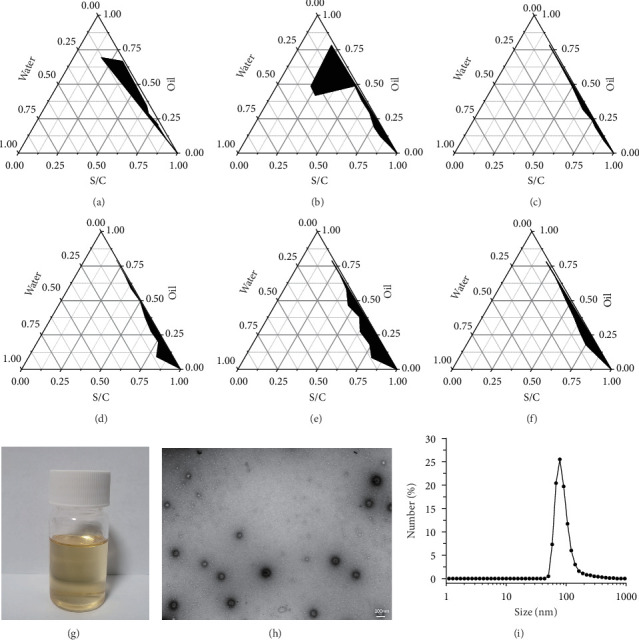
Preparation and characteristics of APSN. (A) The cosurfactant is PEG-400. (B) The cosurfactant is 1,2-propanediol. (C) The cosurfactant is anhydrous ethanol. (D) Km = 2. (E) Km = 1.5. (F) Km = 1. (G) Image of APSN. (H) TEM of APSN. (I) The hydration diameter of APSN was determined by DLS.

A surfactant mixture with a mass ratio of Span 80 to Tween 80 of 2:1 was selected for the nanoemulsion formulation. To determine the optimal cosurfactant, pseudo-ternary phase diagrams were constructed using PEG-400 (Figure 1A), 1,2-propanediol (Figure 1B), and anhydrous ethanol (Figure 1C). Among these, 1,2-propanediol yielded the largest nanoemulsion region (Figure 1B), indicating its superior ability to facilitate nanoemulsion formation compared to PEG-400 and anhydrous ethanol. Therefore, 1,2-propanediol was chosen as the cosurfactant. The influence of the surfactant-to-cosurfactant mass ratio (Km) on nanoemulsion formation was further investigated using pseudo-ternary phase diagrams. Diagrams were constructed for Km values of 2 (Figure 1D), 1.5 (Figure 1E), and 1 (Figure 1F). The largest nanoemulsion area was observed at a Km value of 1.5 (Figure 1E), which was subsequently adopted for the final APSN formulation.

The influence of different concentrations of APS on nanoemulsion formation was also evaluated, with results summarized in Table 1. As shown in Table 1, APS aqueous solutions with concentrations ranging from 1 to 20 mg/mL successfully formed nanoemulsions at 4°C, room temperature, and 37°C. Furthermore, no signs of stratification or demulsification were detected after high-speed centrifugation, suggesting that APS concentrations up to 20 mg/mL did not interfere with nanoemulsion formation. However, at a concentration of 25 mg/mL, nanoemulsion formation was inhibited. Therefore, an APS concentration not exceeding 20 mg/mL was optimal. Based on these experimental results, 20 mg/mL APS aqueous solution was chosen as the aqueous phase for the final nanoemulsion formulation. The resulting nanoemulsion is illustrated in Figure 1G. The physicochemical properties of the optimized APSN formulation were further analyzed. TEM revealed spherical nanoparticles with uniform size distribution (Figure 1H). DLS analysis showed that the hydrated diameter of APSN was approximately 78.82 nm (Figure 1I), and the zeta potential was about −15.6 mV.

### 3.2. Biocompatibility of APSN

**Figure 2:**
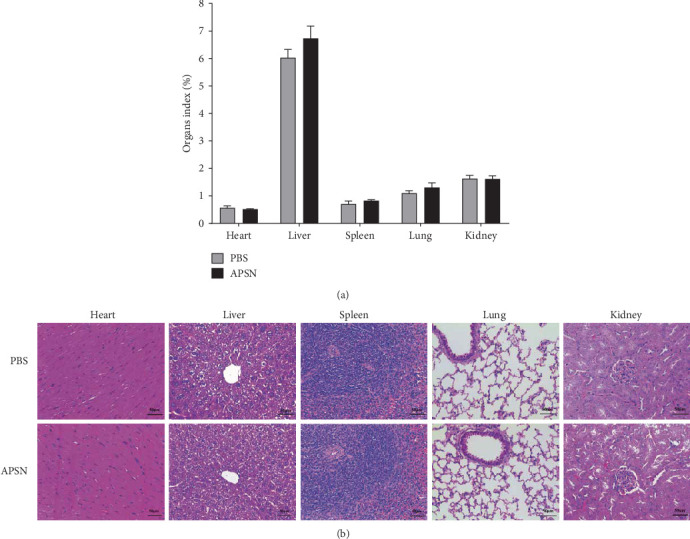
Biocompatibility of APSN. (A) Organ-to-body weight ratios of mice in different immunization groups (PBS and APSN). (B) Histopathological changes in the heart, liver, spleen, lung, and kidney of mice after intramuscular injection of APSN or PBS.

Biocompatibility is a fundamental requirement for vaccine adjuvants. The in vivo biocompatibility of APSN was evaluated in BALB/c mice. Mice were intramuscularly injected with 100 µg of APSN or PBS. Minor increases in certain blood biochemical parameters were observed in the APSN-treated group compare to PBS-immunized group; however, all measured values remained within normal reference ranges, indicating no substantial impact on hematological indices (Table 2). The measure results of the organ-to-body weight ratios of the heart, liver, spleen, lung, and kidney showed no significant difference between the APSN-immunized group and the PBS-immunized group (Figure 2A). Furthermore, histopathological examination of major organs also revealed no notable pathological changes in tissues from APSN-treated mice compared to controls (Figure 2B). The results above collectively demonstrate that APSN has good biocompatibility in vivo.

### 3.3. Stability of APSN and FMDV VLPs–APSN

**Figure 3:**
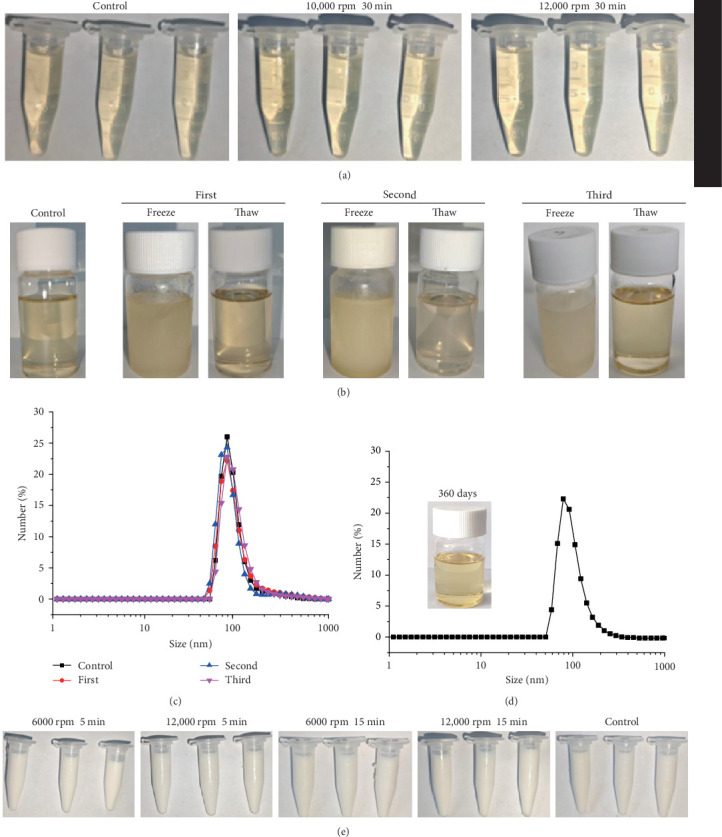
Stability of the APSN adjuvant and APSN–VLPs vaccine. (A) Take photos to observe the morphology of APSN after centrifugation. (B) Take photos to observe the changes of APSN after three repeated freeze–thaw cycles. (C) DLS determined the hydrated particle size of APSN after three repeated freeze–thaw cycles. (D) Visual observation documented the appearance changes of APSN during 360 d storage at room temperature, and DLS determined alterations in hydrated diameter. (E) Take photos to observe the stability of VLPs–APSN after centrifugation.

Nanoemulsion stability is a critical factor for the practical application of vaccine adjuvants. To assess the stability of APSN, the nanoemulsion was subjected to centrifugation at 10,000 and 12,000 rpm for 30 min. Visual inspection revealed no signs of precipitation, flocculation, or phase separation following centrifugation (Figure 3A). Furthermore, after undergoing three freeze–thaw cycles, the APSN nanoemulsion maintained its original appearance without stratification or turbidity (Figure 3B). DLS analysis indicated that the hydrated particle sizes before freeze–thaw and after 1, 2, and 3 freeze–thaw cycles were no significant change (Figure 3C). Visual observation indicated that the APSN appearance remained unchanged after 360 days of storage, showing no signs of precipitation, flocculation, or phase separation; DLS analysis demonstrated no significant change in the hydrated diameter of APSN following 360 days of storage compared with that initially prepared and detected (Figure 3D). The above results indicated that APSN possesses excellent stability, supporting its potential suitability as a vaccine adjuvant.

To analyze the effect of APSN as an adjuvant for the FMDV VLPs vaccine, we prepared FMDV VLPs according to the protocol established in our laboratory (Figure S1). We prepared a nanoemulsion vaccine by adjusting the ratio of FMDV VLPs and APSN. The stability of the VLPs–APSN vaccine formulation was assessed by centrifugation. No delamination or phase separation was observed during this period, indicating good stability of the vaccine formulation (Figure 3E).

### 3.4. Immunogenicity of the FMDV–VLPs–APSN Vaccine in Mice

**Figure 4:**
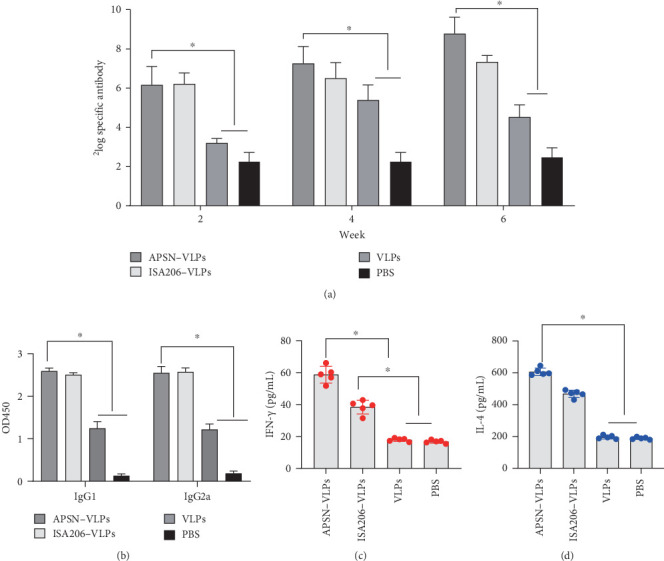
The immune response of mice. (A) Levels of specific antibodies in serum from different immunization groups (PBS, VLPs, ISA206–VLPs, and APSN–VLPs) were measured by ELISA. (B) Levels of IgG1 and IgG2a in serum from different immunization groups were measured by ELISA. (C) The level of IFN-γ in the serum of immunized mice was determined by ELISA. (D) The level of IL-4 in the serum of immunized mice was determined by ELISA. *⁣*^*∗*^ indicates a significant statistical difference *p* < 0.05.

The efficacy of APSN as an adjuvant for the FMDV VLPs vaccine was evaluated in mice. FMDV-specific antibody titers in serum were measured by ELISA. Both APSN–VLPs and the ISA-206–VLPs significantly enhanced antibody level compared to FMDV VLPs alone. Notably, the APSN–VLPs vaccine elicited higher antibody titers compared to the ISA-206–VLPs vaccine (Figure 4A). The IgG subtypes results revealed that both IgG1 and IgG2a levels were significantly elevated in the APSN–VLPs group compared to the VLPs-alone group (Figure 4B). As IgG1 and IgG2a are associated with Th2-type humoral immunity and Th1-type cellular immunity, respectively [28], the results suggested that APSN could enhance humoral and cellular immune responses induced by FMDV VLPs. To elucidate the mechanism by which APSN potentiates FMDV VLPs in eliciting immune responses, we measured the IFN-γ levels in the serum, which are associated with cellular immunity. Moreover, the IL-4 level associated with the humoral immune response was also measured using ELISA [29]. The APSN–VLPs vaccine induced significantly higher levels of IFN-γ (Figure 4C) and IL-4 (Figure 4D) compared to FMDV VLPs alone. Furthermore, IFN-γ levels were significantly higher in the APSN–VLPs group compared to the ISA206–VLPs group (Figure 4C), indicating APSN could assist FMDV VLPs in inducing a stronger cellular immune response. The results demonstrate that APSN effectively enhances both humoral and cellular immune responses to FMDV VLPs in mice, and its ability to promote cellular response appears superior to that of the commercial adjuvant ISA206.

### 3.5. The Results of the Pig Immunization

**Figure 5:**
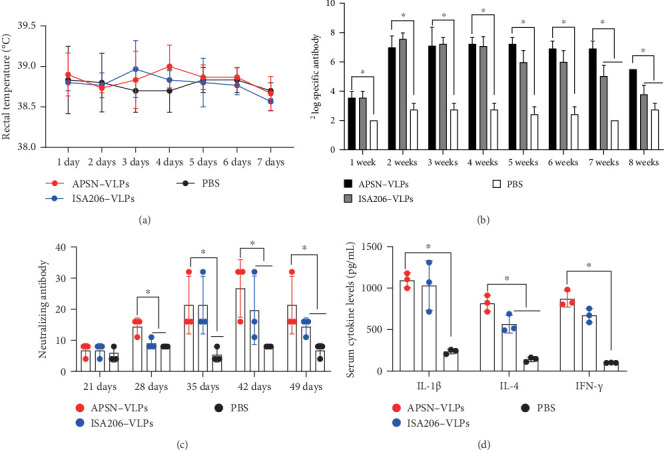
The results of the pig immunization. (A) The body temperature of pigs in different immunization groups. (B) The specific antibody levels of pigs in different immunization groups (PBS, ISA206–VLPs, and APSN–VLPs) detected by ELISA. (C) The neutralizing antibody levels of pigs in different immunization groups were measured by microneutralization test. (D) The cytokine levels (IL-1β, IL-4, and IFN-γ) of pigs in different immunization groups were assay. *⁣*^*∗*^ indicates a significant statistical difference *p* < 0.05.

To further evaluate the efficacy of APSN as an adjuvant for the FMDV VLPs vaccine, we conducted an immunization study on pigs, which are the natural host of FMDV. Body temperature was monitored daily for 7 days postimmunization to assess the safety of the vaccine. No pigs in the APSN-adjuvanted group exhibited a temperature increase greater than 0.5°C, indicating that the vaccine did not induce significant inflammatory reactions (Figure 5A). The results of specific antibody titers result revealed that the APSN–VLPs vaccine induced a longer antibody duration compared to the ISA206–VLPs vaccine (Figure 5B). The neutralizing antibody titer, which is a key indicator of protection against FMDV infection, was also evaluated. The APSN–VLPs group consistently exhibited higher neutralizing antibody titers compared to the ISA206–VLPs group (Figure 5C). Furthermore, the levels of IL-1β, IL-4, and IFN-γ, which are related to the inflammatory response, humoral immune response, and cellular immune response, were detected. The APSN–VLPs vaccine induced higher levels of cytokines (IL-1β, IL-4, and IFN-γ) compared to the ISA206–VLPs group (Figure 5D). The result further confirmed that APSN assists FMDV VLPs could induce better immune effects than the ISA206 adjuvant.

## 4. Discussion

Vaccine immunization is crucial for preventing and controlling infectious disease outbreaks. As an important component of vaccines, adjuvants have the effect of enhancing the immune response induced by antigens [30]. Nanoemulsion adjuvants, owing to their biocompatibility, stability, ability to activate immune responses and to prolong antigen immunity, have been extensively studied and applied [31]. Immunoenhancers could effectively enhance both nonspecific and specific immune responses. In this study, a stable nanoemulsion adjuvant (APSN) was developed using a low-energy emulsification (phase inversion) method. The oil phase consisted of white oil, with Span80 and Tween80 as surfactants, and 1,2-propanediol as a cosurfactant. The precise ratio of these components was determined using a pseudo-ternary phase diagram. APS solution, known for its immune-enhancing properties, was used as the aqueous phase. This method has the advantages of simplicity, low production cost, and scalability for large-scale production.

Emulsion stability is a critical factor for evaluating the efficacy of oil emulsion adjuvants. Particle size, homogeneity, and surface charge are closely linked to emulsion stability [32]. Smaller particle sizes exhibit greater stability compared to larger ones. For instance, MF59, a squalene-based adjuvant used in commercially available influenza vaccines in Europe, has an optimized droplet size of 155–160 nm and demonstrates stability for up to 2 years when stored at 2–8°C [33]. The APSN nanoemulsion developed in this study exhibited a hydrated particle size of 70–90 nm and maintained its particle size after 360 days of storage at room temperature, demonstrating its robust stability. This stability is crucial, as smaller particles are known to facilitate faster movement into the lymphatic system, enhancing their targeting of immune cells [34, 35]. Beyond stability, biocompatibility is a prerequisite for novel vaccine adjuvants. The biocompatibility of APSN was assessed through comprehensive analysis of immunized animals, including observations of mental state, body temperature, biochemical indices, and histological analysis. APSN demonstrated good safety at the immune dose, causing no observable adverse effects.

Research on Chinese medicinal plant polysaccharides has revealed their potential as immune enhancers in vaccines [36, 37]. APS, a prominent example, has been shown to enhance antibody levels and lymphocyte proliferation induced by the Newcastle disease vaccine [38]. Many plant polysaccharides possess amphiphilic groups (e.g., hydroxyl, acetyl, and methoxy groups) that can reduce oil–water interfacial tension and improve emulsion stability [39]. In this study, APS was incorporated as the aqueous phase of the APSN nanoemulsion, contributing to both enhanced emulsion stability and improved immunization efficacy. An ideal adjuvant should regulate both humoral and cellular immunity. Specific antibodies, secreted by differentiated B lymphocytes (plasma cells) upon antigenic stimulation, mediate humoral immunity through neutralization, complement activation, opsonization, and antibody-dependent cell-mediated cytotoxicity. IFN-γ, a key cytokine in cellular immunity, plays a crucial role in antiviral responses and immunomodulation [40]. Our results demonstrate that APSN, as an adjuvant, elevated both specific antibody levels and IFN-γ levels induced by FMDV VLPs, indicating its ability to promote both humoral and cellular immune responses. Furthermore, FMDV VLPs formulated with APSN elicited higher levels of specific and neutralizing antibodies, and cytokines compared to the commercial FMDV adjuvant ISA-206. This enhanced efficacy may be attributed to the ability of APS to activate immune cells and induce cytokine secretion, although the precise mechanisms warrant further investigation.

## 5. Conclusion

The nanoemulsion adjuvant of APSN developed in this study demonstrated good stability and biocompatibility. Furthermore, APSN effectively enhanced cellular immune responses and humoral immune responses induced by FMDV VLPs, conferring robust protection against FMDV challenge. Notably, APSN outperformed the commercial adjuvant ISA-206, which indicates that APSN has the potential to become a highly promising adjuvant for veterinary vaccines.

## Figures and Tables

**Table 1:** Effect of different APS concentrations on the formation of nanoemulsion system.

APS concentration (%)	4°C	37°C	Room temperature	Result determination
1 mg/mL	Clear emulsion	Clear emulsion	Clear emulsion	+
5 mg/mL	Clear emulsion	Clear emulsion	Clear emulsion	+
10 mg/mL	Clear emulsion	Clear emulsion	Clear emulsion	+
15 mg/mL	Clear emulsion	Clear emulsion	Clear emulsion	+
20 mg/mL	Clear emulsion	Clear emulsion	Clear emulsion	+
25 mg/mL	Clear emulsion	Clear emusion	Clear emulsion	×

*Note:* “+” indicates nanoemulsion formation; “×” indicates the absence of nanoemulsion formation. The mass ratio of mixed surfactants Tween 80:Span 80 (m:m) = 2:1; The cosurfactant is 1,2-propanediol (Km = 1.5). The oil phase is pharmaceutical-grade white oil. APS is an astragalus polysaccharide aqueous solution.

**Table 2:** Biochemical indices of immunized mice.

Groups	Test items	Test values	Reference value
APSN	Aspartate aminotransferase	138.7	60.0–220.0
	Creatine kinase	841.2	5.0–1000.0
	Creatinine	25.6	22.0–97.0
	Total bilirubin	14.94	2.00–20.00

PBS	Aspartate aminotransferase	98.2	60.0–220.0
	Creatine kinase	443.7	5.0–1000.0
	Creatinine	24.3	22.0–97.0
	Total bilirubin	11.05	2.00–20.00

## Data Availability

The data that support the findings of this study are available from the corresponding author upon reasonable request.

## Supporting Information

Supporting Information Figure S1. Preparation of FMDV VLPs. The purified FMDV structural proteins and the FMDV structural proteins digested by Sumo enzyme were analyzed by (A) SDS–PAGE and (B) western blot. (C) Hydrodynamic diameter of the FMDV VLPs was measured by DLS.
