# Supplementary material for: Astragalus Polysaccharide Nanoemulsion: A Promising Adjuvant for Foot-And-Mouth Disease Virus-Like Particle Vaccines
Source: Transbound Emerg Dis. 2025 Sep 25;2025:6693841. doi: 10.1155/tbed/6693841 (PMC12490925; doi:10.1155/tbed/6693841)
Supplement: Supporting Information — Figure S1. Preparation of FMDV VLPs. The purified FMDV structural proteins and the FMDV structural proteins digested by Sumo enzyme were analyzed by (A) SDS–PAGE and (B) western blot. (C) Hydrodynamic diameter of the FMDV VLPs was measured by DLS. [file 6693841.f1.docx]

**Astragalus Polysaccharide Nanoemulsion: A Promising Adjuvant for Foot-and-Mouth Disease Virus-Like Particle Vaccines**

Xiaoni Shi^1,2#^，Zhidong Teng^1#^，Kun Yang^1^, Hetao Song^1^, Yun Zhang^1^, Shuzhen Tan^1^,  Hu Dong^1^, Shiqi Sun^1^, Yaozhong Ding^1^, Huichen Guo^1,3,4*^

^1^State Key Laboratory for Animal Disease Control and Prevention, College of Veterinary Medicine, Lanzhou University, Lanzhou Veterinary Research Institute, Chinese Academy of Agricultural Sciences, Lanzhou, China

^2^School of Chemical Engineering, Lanzhou City University, Lanzhou, 730070, China

^3^College of Veterinary Medicine, Gansu Agricultural University, Lanzhou, China

^4^Yunnan Tropical and Subtropical Animal Virus Diseases Laboratory, Yunnan Animal Science and Veterinary Institute, Kunming, Yunnan, China

**1. Preparation of the FMDV-VLPs**

Foot-and-mouth disease virus (FMDV) structural proteins with a His-Sumo tag were expressed and purified using Ni²⁺ affinity chromatography. The His-Sumo tag was subsequently cleaved using Sumo protease and the structural proteins (VP0, VP1, and VP3) of FMDV were obtained (Figure**S1A** and **S1B**). The purified structural proteins were assembled in the assembly buffer. Dynamic light scatterer (DLS) analysis revealed that the assembled sample had an average hydrated diameter of approximately 45 nm, indicating that the FMDV VLPs were successfully obtained (Figure **S1C**).


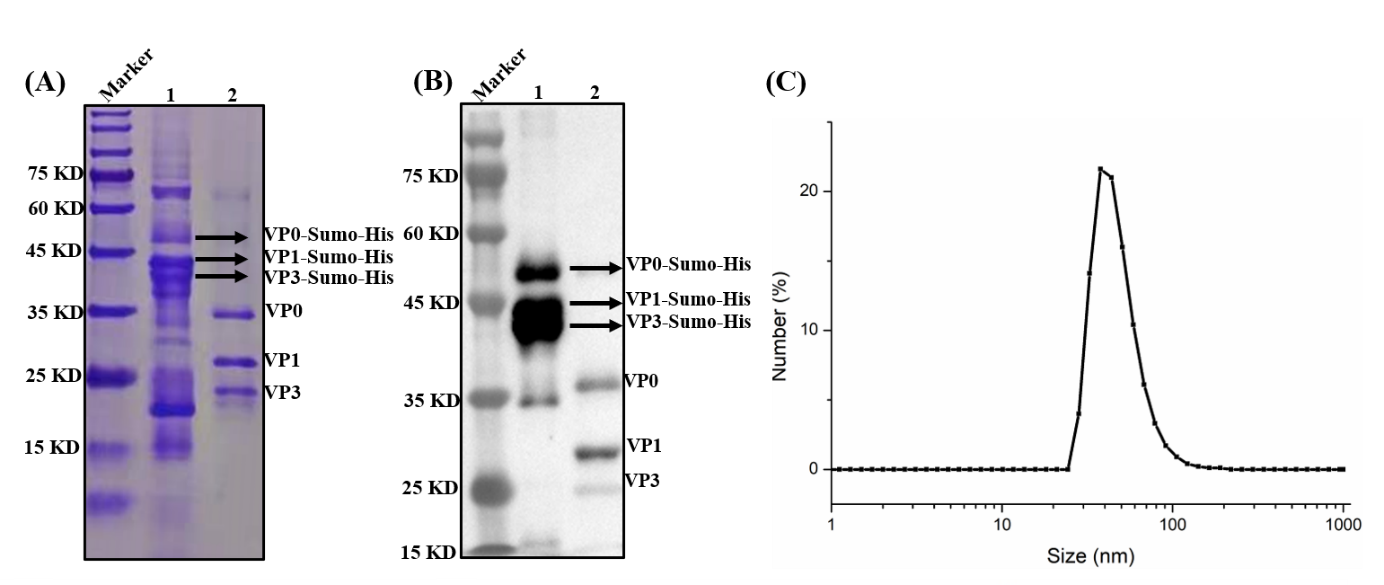


Figure S1: Preparation of FMDV-VLPs

The purified FMDV structural proteins and the FMDV structural proteins digested by Sumo enzyme were analyzed by (A) SDS-PAGE and (B) Western Blot; (C) Hydrodynamic diameter of the FMDV VLPs was measured by DLS.
